# Acute Onset of Remitting Seronegative Symmetrical Synovitis With Pitting Edema (RS3PE) Two Weeks After COVID-19 Vaccination With mRNA-1273 With Possible Activation of Parvovirus B19: A Case Report With Literature Review

**DOI:** 10.7759/cureus.24952

**Published:** 2022-05-12

**Authors:** Hiroto Arino, Naokazu Muramae, Mitsumasa Okano, Kenta Mori, Kazunori Otsui, Kazuhiko Sakaguchi

**Affiliations:** 1 Department of General Internal Medicine, Kobe City Medical Center General Hospital, Kobe, JPN; 2 Department of General Internal Medicine, Kobe University Graduate School of Medicine, Kobe, JPN; 3 Department of General Internal Medicine, Kobe University Hospital, Kobe, JPN

**Keywords:** parvovirus b19, peripheral edema, mrna-1273 (moderna), sars-cov-2 vaccination, rs3pe

## Abstract

Remitting seronegative symmetrical synovitis with pitting edema (RS3PE) is a rare clinical entity characterized by “remitting,” “seronegative,” and “symmetrical” synovitis with pitting edema on the dorsum of the hands and feet. Although rheumatic or malignant diseases are diseases that are known to coexist with RS3PE, other factors such as medication, infection, and vaccination have been reported to be associated with RS3PE. Here, we present a case of RS3PE syndrome that satisfied all four diagnostic criteria of RS3PE (pitting edema in the limbs, acute onset, age ≥ 50 years, and/or rheumatoid factor negativity) after mRNA-1273 SARS-CoV-2 vaccination.

## Introduction

Vaccination against SARS-CoV-2 infection is increasing worldwide due to the pandemic [1,2]. BNT162b2 (Pfizer), mRNA-1273 (Moderna), and ChAdOx1 nCoV-19 (AstraZeneca) vaccines are available in Japan. Their efficacy in preventing severe respiratory conditions has been established [3-5]. However, as the number of vaccinated people increased, it became evident that various adverse reactions such as fever, myalgia, general fatigue, skin lesions, thrombosis, and deterioration of preexisting rheumatic disease could occur as a result of vaccination [2].

Remitting seronegative symmetrical synovitis with pitting edema (RS3PE) syndrome is a rare clinical condition that usually occurs in elderly people, and it is characterized by an acute onset of symptoms, rheumatoid factor negativity, presence of symmetrical peripheral joint pain, and peripheral pitting edema [6-8]. Although the etiology is not fully understood, rheumatic, malignant, and infectious diseases have been reported to coexist with RS3PE. In addition, some medications and vaccines have been reported to cause it [9-11].

Here, we report a case of R3SPE syndrome associated with COVID-19 mRNA-1273 vaccine inoculation to help general physicians correctly diagnose and treat it.

## Case presentation

A 65-year-old male was referred to our hospital on account of prolonged fever, bilateral peripheral edema, polyarthralgia, and difficulty standing and walking. His symptoms developed two weeks after he was administered the second dose of mRNA-1273 SARS-CoV-2 vaccination. Since his symptoms were considered to be related to an infectious disease, he was treated with nonsteroidal anti-inflammatory drugs and antibiotics for two weeks at a previous general medicine clinic; however, his symptoms did not improve. His medical history included angina pectoris, recurrent urinary tract infection, and urosepsis. There was no history of tuberculosis. He had not drunk alcohol for 1-2 years, smoked cigarettes (40 pack-years), and had no family history of collagen disease.

**Figure 1:**
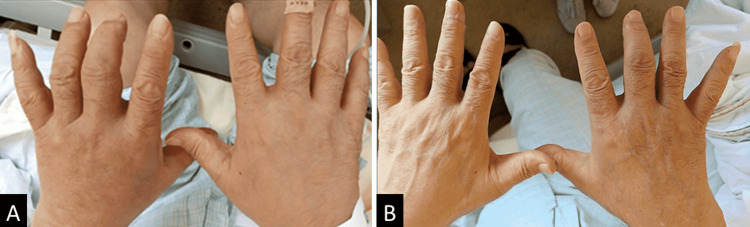
Photographs of both hands A: Polyarthritis and pitting edema in both hands and wrists on admission. B: Arthritis and edema dramatically and immediately improved after corticosteroid therapy.

His vital signs were stable, except for his body temperature (38.1°C). On physical examination, tenderness and pitting edema in both wrists and distal ankle joints were evident. The metacarpophalangeal (MCP) and proximal interphalangeal (PIP) joints of the right third and fourth fingers were swollen with accompanying mild redness and warmth (Figure 1A). The active and passive ranges of motion of his shoulder joints were limited to 60° abduction due to pain. He could not walk due to the pain in his ankle joints. There were no findings suggestive of meningitis, edema in the orbit, Gottron’s sign in the fingers, or abnormal findings in the truncal skin.

Laboratory data on admission revealed a marked elevation in C-reactive protein (CRP) levels with mild leukocytosis and anemia (Table 1). Serum levels of matrix metalloproteinase-3 (MMP-3) were also elevated. The PCR test was performed to confirm that the patient’s symptoms were not based on SARS-CoV-2 infection, with negative results. Unfortunately, the serologic test was not performed. Rheumatoid factor, proteinase 3-antineutrophil cytoplasmic antibody (PR3-ANCA), anti-myeloperoxidase-ANCA (MPO-ANCA), and anti-cyclic citrullinated peptide antibodies were negative. Antinuclear antibody was very weakly positive, with a titer of 80 (with a speckled pattern). Hepatitis B surface antibody and antigen and hepatitis C virus antibody tests were negative. Tuberculosis-specific interferon-gamma release assay results were also negative. Both IgG and IgM anti-parvovirus B19 antibodies were positive, with indexes of 8.00 and 4.19, respectively.

**Table 1:** Laboratory data on admission Seg: segmented neutrophil, Eosi: eosinophil, Baso: basophil, Mono: monocyte, Lymp: lymphocyte, TP: total protein, Alb: albumin, T-Bil: total bilirubin, AST: aspartate aminotransferase, ALT: alanine aminotransferase, γ-GTP: γ-glutamyl transferase, LDH: lactate dehydrogenase, BUN: blood urea nitrogen, Cre: creatinine, Glu: glucose, HbA1c: hemoglobin A1c, CPR: C-reactive protein, MMP-3: matrix metalloproteinase-3

Test	Result	Unit	Range
WBC	14,000	/μL	3,300-8,600
Seg	78	%	38-58
Eosi	3	%	0-5
Baso	1		0-1
Mono	8	%	2-8
Lymp	9	%	26-47
RBC	342	×10^4^/μL	386-492
Hb	10.5	g/dL	11.6-14.8
Plt	54.7	×10^4^/μL	15.8-34.8
TP	6.4	g/dL	6.6-8.1
Alb	2.5	g/dL	4.1-5.1
AST	33	IU/L	13-30
ALT	35	IU/L	7-23
γ-GTP	175	IU/L	9-32
LDH	163	IU/L	124-222
T-Bil	0.5	mg/dL	0.4-1.5
BUN	16.7	mg/dL	8-20
Cre	0.81	mg/dL	0.46-0.79
Glu	105	mg/dL	73-109
HbA1c	5.7	%	4.6-6.2
CRP	26.93	mg/dL	-0.14
MMP-3	429	ng/mL	36.9-121

At consulting a rheumatologist, the differential diagnoses included arthritis after parvovirus infection, elderly onset rheumatoid arthritis (EORA), polymyalgia rheumatica (PMR), and crystal-induced arthritis such as gout and calcium pyrophosphate dihydrate deposition disease.

**Figure 2:**
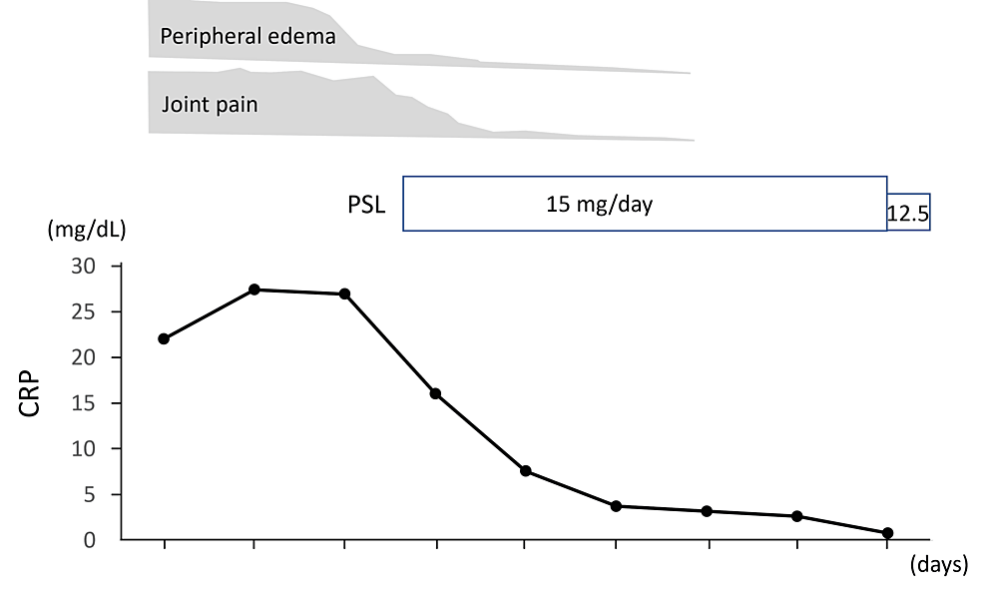
Clinical course of this case Low-dose prednisolone (15 mg/day) dramatically improved his symptoms and C-reactive protein levels.

After confirming a negative blood culture, prednisolone was initiated at a dose of 15 mg/day two days after admission. After the initiation of prednisolone administration, the patient’s edema and joint pain improved dramatically (Figure 1B) and immediately, and serum CRP levels showed an improving trend (Figure 2). The patient was discharged on the 18th day after admission and switched to outpatient care. Corticosteroid therapy was tapered when the serum CRP became negative.

## Discussion

Herein, we presented a corticosteroid-effective case of RS3PE after SARS-CoV-2 vaccination, which did not directly prove the involvement of anti-SARS-CoV-2 antibodies but instead demonstrated the activation of parvovirus B19.

The differential diagnosis of this case was as follows. Arthritis caused by parvoviruses presents with acute, symmetrical arthritis of the joints, wrists, knees, and feet [12,13]. However, 75% of these patients will have a skin rash, and 20% will have a typical slapped cheek appearance [14]. Moreover, the joint symptoms often improve within 1-2 weeks [15]. In our case, because of the absence of erythema, prolonged course despite the use of NSAIDs, and absence of a transient hematopoietic disorder, parvovirus-induced arthritis was less likely. EORA was also considered; however, autoantibodies were negative, there was no destruction of the joints, and pitting edema is not a typical symptom of rheumatoid arthritis. In polyarthritis rheumatica (PMR), both peripheral arthritis and pitting edema are also rare. Crystal-induced arthritis, such as gout, was also less likely because the duration of the symptoms was not compatible. Based on the presence of all four diagnostic criteria (pitting edema in the limbs, acute onset, age ≥ 50 years, and rheumatoid factor negativity), our final diagnosis was RS3PE. Additionally, since rheumatic disease and malignancy are less likely to coexist, the mRNA-1273 SARS-CoV-2 vaccination was suspected to have triggered RS3PE in this case. Although the precise etiology of RS3PE is unknown, it has been reported to be related to malignancy [8], parvovirus infection [16], BCG vaccination [11], and immunotherapies [17]. Recently, two cases of new-onset RS3PE after BNT162b2 mRNA COVID-19 vaccination were reported [9,10]. In one of the cases, antibodies against parvovirus B19 were examined, and the result was negative.

Interestingly, parvovirus infection has been reported to cause RS3PE, although the precise mechanism is unknown [16,18]. In our case, both IgG and IgM for parvovirus were positive on admission, and the same IgM index (4.95) and IgG index (8.93) for parvovirus B19 persisted at six months after initial presentation, suggesting that parvovirus reactivation or persistent infection had occurred.

Although parvovirus reactivation is uncommon, known risk factors include congenital immunodeficiency, HIV infection [19], leukemia, lymphoma, and the use of immunosuppressants [20,21]. However, this patient did not have these risk factors, and the only factor that could have triggered an immune response before the onset of RS3PE syndrome was the mRNA-1273 SARS-CoV-2 vaccination. Viral reactivation after COVID-19 vaccination has been reported for herpes zoster virus, and the cause is suspected to be transient T-lymphocyte depletion after vaccination [22,23]. Transient T-lymphocyte depletion has been reported with ChAdOx1 nCoV-19 [24] and BNT162b1 [25] vaccination, but not with mRNA-1273 vaccination. However, the lymphocytes in this patient were lower than the lower reference value; this might have been involved in the pathogenesis of parvovirus reactivation. After excluding other diseases or conditions, we presumed that parvovirus reactivation triggered by mRNA-1273 SARS-CoV-2 vaccination was the possible mechanism for RS3PE syndrome in this case. To the best of our knowledge, this is the first report of a parvovirus reactivation following mRNA-1273 SARS-CoV-2 vaccination.

In our case, the administration of prednisolone immediately improved his symptoms and serum CRP level. In contrast, malignancy-associated RS3PE syndrome responds poorly to corticosteroid therapy [26]. Continuous screening for malignancy may be necessary, even after remission of RS3PE [27].

## Conclusions

Our case highlights the importance of clinical suspicion of RS3PE, a rare etiology and not fully understood disorder, after SARS-CoV-2 vaccination, although direct proof of the involvement of anti-SARS-CoV-2 antibody was lacking. Since prompt diagnosis and immediate initiation of corticosteroids improved the patient’s symptoms, it is important for clinicians to keep in mind the possibility that rare phenomena may occur after vaccination. Further accumulation of cases is awaited.
